# Chronic obstructive pulmonary disease and influenza vaccination effect in preventing outpatient and inpatient influenza cases

**DOI:** 10.1038/s41598-022-08952-0

**Published:** 2022-03-22

**Authors:** Iván Martínez-Baz, Itziar Casado, Ana Navascués, María Eugenia Portillo, Marcela Guevara, Carmen Ezpeleta, Jesús Castilla

**Affiliations:** 1 Instituto de Salud Pública de Navarra, Leyre 15, 31003 Pamplona, Spain; 2 CIBER Epidemiología y Salud Pública (CIBERESP), Pamplona, Spain; 3 Navarra Institute for Health Research (IdiSNA), Pamplona, Spain; 4 Clinical Microbiology Department, Complejo Hospitalario de Navarra, Pamplona, Spain

**Keywords:** Immunology, Microbiology, Diseases, Health care, Medical research

## Abstract

Evidence of influenza vaccine effectiveness in preventing confirmed influenza among persons diagnosed with chronic obstructive pulmonary disease (COPD) is scarce. We assessed the average effect of influenza vaccination in the current and prior seasons in preventing laboratory-confirmed influenza in COPD patients. We carried out a pooled test-negative case–control design in COPD patients hospitalized or presented to primary healthcare centres with influenza-like illness who were tested for influenza in 2015/2016 to 2019/2020 seasons in Navarre, Spain. Influenza vaccination status in the current and 5 prior seasons was compared between confirmed-influenza cases and test-negative controls. Vaccination effect was compared between target patients for vaccination with and without COPD. Out of 1761 COPD patients tested, 542 (31%) were confirmed for influenza and 1219 were test-negative controls. Average effect for current-season vaccination in preventing influenza was 40% (95% CI 20–54%), and for vaccination in prior seasons only was 24% (95% CI –10 to 47%). Point estimates seemed higher in preventing outpatient cases (60% and 58%, respectively) than inpatient cases (37% and 19%, respectively), but differences were no statistically significant. Influenza vaccination effect was similar in target population with and without COPD (*p* = 0.339). Influenza vaccination coverage in control patients with COPD was 68.3%. A 13.7% of the influenza cases in patients with COPD could be prevented by extending the influenza vaccine coverage. Average effect of current-season influenza vaccination was moderate to prevent influenza in COPD persons. The increase of influenza vaccination coverage can still prevent COPD exacerbations.

## Introduction

Chronic obstructive pulmonary disease (COPD) is a highly prevalent condition and one of the main causes of years of life lost^1^. Acute respiratory infections, especially influenza, are frequently causes of the exacerbation of COPD^2,3^. Vaccination is the main preventive measure against influenza and its complications^4^. Since patients with COPD are at increased risk of hospitalization for influenza infections, annual influenza vaccination is recommended worldwide for them^4–6^. However, the protective serum antibody response could be suboptimal in patients with COPD^7,8^.

Influenza vaccine effectiveness in people with COPD was evaluated in observational studies against non-specific endpoints such as hospitalizations or deaths by all causes^9–13^. These studies are prone to relevant bias because influenza virus infection is clinically indistinguishable from other respiratory infections not preventable by influenza vaccination^9,11–13^. The scarce evidence among persons with COPD has estimated that influenza vaccination reduced around 38–43% confirmed-influenza hospitalizations^14–16^. However, none of these studies estimated influenza vaccine effectiveness in the general practice and hospital settings, and neither had considered the effect of vaccination history, although patients with COPD frequently accumulate several influenza vaccines over years and prior vaccination has shown a relevant role in other chronic conditions^17^. A complete overview of the influenza vaccination effect (IVE) would require considering both settings and vaccination history.

The objective of this study was to evaluate the average effect and impact over five seasons of the influenza vaccination status in the current and prior seasons in preventing laboratory-confirmed influenza in persons with COPD. We also compared these estimates against those from the target population for influenza vaccination without COPD diagnosis.

## Methods

### Study population

This test-negative case–control study was performed in the region of Navarre, Spain, where annual IVE studies have been conducted since 2009^18–24^. The Navarre Health Service provides universal health care. The trivalent inactivated influenza vaccine was annually recommended and offered free of charge to the target population, which included people aged 60 years and more or with major chronic conditions, including all patients with COPD regardless of their age.

Influenza surveillance was based on automatic reporting of cases of medically-attended influenza-like illness (ILI) from all primary healthcare centres and hospitals^21^. ILI was defined as the sudden onset of any general symptom (fever or feverishness, malaise, headache or myalgia) in addition to any respiratory symptom (cough, sore throat or shortness of breath)^25^. The protocol for influenza cases in hospitals established early detection and double swabbing, nasopharyngeal and pharyngeal, at admission of all hospitalized patients with ILI, regardless of the cause of admission or any other clinical manifestation. A sentinel network composed of a representative sample of 16% of primary healthcare physicians was trained to perform a double swab, after verbal informed consent, from all patients diagnosed with ILI and whose symptoms had begun within the previous 5 days. Swabs were tested for influenza virus by reverse-transcription polymerase-chain-reaction (RT-PCR).

In every influenza vaccination campaign, diagnoses of COPD and other major chronic conditions (diabetes mellitus, cardiovascular disease, asthma, renal disease, cancer, liver cirrhosis, dementia, stroke, immunodeficiency, rheumatic disease and severe obesity) were obtained from the electronic medical records of primary healthcare. These conditions are codified according to the International Classification of Primary Care, Second Edition, being R95 the code of COPD diagnosis^26^.

Influenza vaccination status in the current and 5 previous seasons and the pneumococcal vaccination status were obtained from the regional vaccination register^27^.

### Study design

A test-negative case–control study^28^, nested in the cohort of population covered by the Navarre Health Service, was carried out including patients with continuous residence in the region during the 5 years before the analysed influenza season. The study pooled over five influenza seasons (2015–2016 to 2019–2020) patients aged 25 years and over who were hospitalized or presented to primary healthcare centres with ILI and who were tested for influenza virus by RT-PCR, regardless of the presence of other clinical manifestations. Healthcare workers and nursing homes residents were excluded to prevent bias, since they have a different use of health services.

This study evaluated the average effect over five seasons of the influenza vaccination status in the current and prior seasons, regardless of variations in the effectiveness from season to season. The primary analysis evaluated IVE in preventing influenza in patients with COPD. Cases were COPD patients with confirmed influenza virus by RT-PCR, and controls were COPD patients who tested negative for any influenza virus.

The test-negative case–control design was again applied to the whole influenza vaccination target population to compare IVE estimates between persons with and without COPD diagnosis given a similar influenza vaccination status.

All data were treated in a strictly confidential manner according to the ethical principles of the Helsinki Declaration of 1964 revised by the World Medical Organization in Fortaleza 2013, and the Spanish and European personal data protection regulations. The Navarra Ethical Committee for Medical Research approved the study protocol and waived the requirement of obtaining signed consent from patients (approval codes: 85/11, 2015/95 and 2017/88). Analyses were performed with anonymous data.

### Statistical analyses

Characteristics of cases and controls were compared by χ^2^ test. Logistic regression models to obtain adjusted odds ratios (aOR) and their 95% confidence intervals (95% CI). Models were adjusted for age groups (25–64, 65–84 and ≥ 85 years), other major chronic conditions, healthcare setting (primary healthcare or hospital), and month-season of sample collection. Sensitivity analyses were performed for additional adjustment for sex, pneumococcal vaccination status, number of outpatient visits in the prior year and hospitalization in the prior 12 months. The IVE was estimated as (1 – aOR) × 100%.

Firstly, IVE estimates were obtained for combinations of the influenza vaccination status in the current and prior seasons, using people unvaccinated in the current and prior seasons as the reference category. When differences among category estimates were not statistically significant, these categories were aggregated to simplify the presentation of results as follows: current-season vaccination regardless of prior doses, vaccination in prior seasons but not in the current one, and neither current-season vaccination nor prior doses as the reference category^17^. The rationale for this categorization has been described elsewhere^29^.

Among people vaccinated in prior seasons, the incremental effect of current season vaccination was also estimated. The interaction terms between the vaccination status and sex, age group, diagnosis of asthma or healthcare setting were tested. Stratified analyses were carried out by sex, age group (25–64 and ≥ 65 years), presence of other major chronic conditions, diagnosis of asthma, healthcare setting, influenza season and virus (sub)type (A/H1N1, A/H3N2 and B). Seasons with a minimal circulation of a given (sub)type were excluded from the pooled analysis for that outcome.

Another logistic regression model analysed all the target population for influenza vaccination to compare the IVE estimates between patients with COPD and the rest of the target population for influenza vaccination.

Point estimates of IVE and vaccination coverage were used to assess the impact of received vaccines in the study population. The impact of extending the influenza vaccination coverage up to 100% was estimated by applying the estimates of the IVE to the unvaccinated cases in the current season included in the study.

## Results

### Characteristics of COPD patients

A total of 1761 COPD patients with ILI were tested during the five influenza seasons, 1597 inpatients and 164 outpatients. Influenza virus was confirmed in 542 (31%) patients: 231 A/H3N2, 188 A/H1N1, 119 influenza B, two A not subtyped, one coinfected by influenza A/H3N2 and A/H1N1, and another coinfected by influenza A/H3N2 and B. These influenza cases were compared against 1219 test-negative controls.

In comparison with test-negative controls, influenza cases less frequently had diabetes (27% vs. 32%, *p* = 0.027) and cardiovascular disease (33% vs. 40%, *p* = 0.010), and were males (67% vs. 72%, *p* = 0.029) and younger (*p* = 0.047). No other comorbidities presented statistically significant differences. Among the influenza cases, 69% had been vaccinated against influenza in any of the 5 prior seasons and 57% had received the current season vaccine in comparison to 79% and 68% of the test-negative controls, respectively (*p* < 0.001 for both comparisons) (Table 1).

**Table 1:** Baseline characteristics of individuals with chronic obstructive pulmonary disease consulting or hospitalized for influenza-like illness according to the result of the influenza test.

	Laboratory-confirmed influenza cases n (%)	Influenza-negative controls n (%)	*p* value
Total	542 (100)	1219 (100)	
**Age groups (years)**			0.047
25–64	143 (26)	261 (21)	
65–84	313 (58)	729 (60)	
≥ 85	86 (16)	229 (19)	
**Sex**			0.029
Male	362 (67)	877 (72)	
Female	180 (33)	342 (28)	
**Healthcare setting**			< 0.001
Primary healthcare	92 (17)	72 (6)	
Hospital	450 (83)	1147 (94)	
**Other major chronic conditions**			
Diabetes	147 (27)	395 (32)	0.027
Cancer	148 (27)	367 (30)	0.233
Liver cirrhosis	34 (6)	94 (8)	0.283
Renal disease	91 (17)	228 (19)	0.336
Immunodeficiency	16 (3)	32 (3)	0.697
Asthma	50 (9)	90 (7)	0.187
Cardiovascular disease	180 (33)	483 (40)	0.010
Dementia	10 (2)	38 (3)	0.130
Stroke	38 (7)	95 (8)	0.566
Rheumatic disease	19 (4)	33 (3)	0.361
Severe obesity	25 (5)	50 (4)	0.624
**Vaccination in the current season**			< 0.001
No	233 (43)	387 (32)	
Yes	309 (57)	832 (68)	
**Influenza vaccines in the 5 prior seasons**			< 0.001
0	166 (31)	251 (21)	
1–2	69 (13)	146 (12)	
3–5	307 (56)	822 (67)	
**Influenza season**			< 0.001
2015–2016	80 (14)	150 (12)	
2016–2017	102 (19)	181 (15)	
2017–2018	161 (30)	242 (20)	
2018–2019	123 (23)	288 (24)	
2019–2020	76 (14)	358 (29)	

### Influenza vaccination effect in patients with COPD

We evaluated the effect of combinations of the influenza vaccination status in the current and prior seasons in a pooled analysis of the five influenza seasons. We aggregated categories of influenza vaccination in the current season with and without prior doses when their estimates were not statistically different (Supplementary Tables 1 and 2). On average of the five seasons, the preventive effect against influenza was 40% (95% CI 20–54%) in persons vaccinated in the current season regardless of prior doses, and 24% (95% CI –10 to 47%) in persons vaccinated in prior seasons only, in comparison to unvaccinated people in the current and 5 prior seasons (Table 2). Among people vaccinated in prior seasons, current season vaccination appears to improve the preventive effect (20%; 95% CI –10 to 42%), although the incremental effect did not reach statistical significance (Supplementary Table 3).

**Table 2:** Effect of influenza vaccination status in the current and 5 prior seasons in preventing laboratory-confirmed influenza among patients with chronic obstructive pulmonary disease.

	Cases/controls	Vaccination effect, (95% CI)^a^	*p* value
**All patients**			
Never vaccinated	154/215	Reference	
Vaccination in prior seasons only	79/172	24 (–10 to 47)	0.146
Current season vaccination	309/832	40 (20 to 54)	< 0.001
**Aged 25–64 years**			
Never vaccinated	80/90	Reference	
Vaccination in prior seasons only	25/45	–2 (–98 to 48)	0.965
Current season vaccination	38/126	56 (24 to 74)	0.003
**Aged ≥ 65 years**			
Never vaccinated	74/125	Reference	
Vaccination in prior seasons only	54/127	28 (–13 to 55)	0.150
Current season vaccination	271/706	33 (6 to 52)	0.019
**Male**			
Never vaccinated	96/142	Reference	
Vaccination in prior seasons only	50/116	26 (–18 to 53)	0.212
Current season vaccination	216/619	36 (8 to 55)	0.014
**Female**			
Never vaccinated	58/73	Reference	
Vaccination in prior seasons only	29/56	25 (–41 to 60)	0.375
Current season vaccination	93/213	43 (7 to 64)	0.023
**Primary healthcare patients**			
Never vaccinated	54/30	Reference	
Vaccination in prior seasons only	5/7	58 (–85 to 92)	0.252
Current season vaccination	33/35	60 (–5 to 85)	0.061
**Hospital patients**			
Never vaccinated	100/185	Reference	
Vaccination in prior seasons only	74/165	19 (–19 to 45)	0.285
Current season vaccination	276/797	37 (15 to 53)	0.002

*CI* confidence interval.

^a^Vaccination effect adjusted by age groups (25–64, 65–84 and ≥ 85 years), other major chronic conditions, healthcare setting (primary healthcare or hospital), and month-season of sample collection.

In the sensitivity analysis additionally adjusted for sex, pneumococcal vaccination status, number of outpatient visits in the prior year and hospitalization within the prior 12 months, the estimated IVE remained with no relevant changes (Supplementary Table 4).

The IVE point estimate for current-season vaccination seemed higher in outpatients (60%; 95% CI – 5 to 85%) in comparison to inpatients (37%; 95% CI 15–53%, *p*_interaction_ = 0.552), in subjects aged 25–64 years in comparison to those aged 65 years and over (56% vs. 33%; *p*_interaction_ = 0.177), in females than in males (43% vs. 36%; *p*_interaction_ = 0.897), and in patients with co-diagnosis of asthma as compared to those without asthma (67% vs. 39%; *p*_interaction_ = 0.854), but all these comparisons were no statistical significant. In COPD patients with other comorbidities, the protective effect of current-season vaccination was 42% (95% CI 20–58%), similar to the overall analysis result (Tables 2, 3).

**Table 3:** Effect of influenza vaccination status in the current and 5 prior seasons in preventing laboratory-confirmed influenza among patients with chronic obstructive pulmonary disease by (sub)type and presence of other major chronic conditions.

	Cases/controls	Vaccination effect, % (95% CI)^a^	*p* value
**A/H1N1 subtype**^**b**^			
Never vaccinated	67/182	Reference	
Vaccination in prior seasons only	20/135	47 (5 to 71)	0.034
Current season vaccination	102/721	49 (23 to 66)	0.001
**A/H3N2 subtype **^**c**^			
Never vaccinated	56/158	Reference	
Vaccination in prior seasons only	37/124	6 (–61 to 44)	0.830
Current season vaccination	139/579	28 (–8 to 52)	0.116
**B type **^**d**^			
Never vaccinated	32/127	Reference	
Vaccination in prior seasons only	21/106	6 (–93 to 54)	0.867
Current season vaccination	66/517	52 (14 to 73)	0.013
**Codiagnosis of asthma**			
Never vaccinated	14/11	Reference	
Vaccination in prior seasons only	6/15	46 (–196 to 90)	0.473
Current season vaccination	30/64	67 (–13 to 90)	0.077
**No codiagnosis of asthma**			
Never vaccinated	140/204	Reference	
Vaccination in prior seasons only	73/157	22 (–14 to 47)	0.198
Current season vaccination	279/768	39 (19 to 54)	0.001
**Patients with other major chronic conditions**			
Never vaccinated	93/143	Reference	
Vaccination in prior seasons only	63/129	21 (–20 to 48)	0.271
Current season vaccination	243/675	42 (20 to 58)	0.001
**Patients without other major chronic conditions**			
Never vaccinated	61/72	Reference	
Vaccination in prior seasons only	16/43	31 (–51 to 68)	0.353
Current season vaccination	66/157	26 (–30 to 58)	0.297

*CI* confidence interval.

^a^Vaccination effect adjusted by age groups (25–64, 65–84 and ≥ 85 years), other major chronic conditions, healthcare setting (primary healthcare or hospital), and month-season of sample collection.

^b^Pooled A/H1N1 analysis includes 2015–2016, 2017–2018, 2018–2019 and 2019–2020 seasons.

^c^Pooled A/H3N2 analysis includes 2015–2016, 2016–2017, 2017–2018 and 2018–2019 seasons.

^d^Pooled B analysis includes 2015–2016, 2017–2018 and 2019–2020 seasons.

The average effect of current-season vaccination was moderate in preventing influenza B (52%; 95% CI 14–73%) and influenza A/H1N1 (49%; 95% CI 23–66%), and this effect was low against influenza A/H3N2 (28%; 95% CI – 8 to 52%). Vaccination in previous seasons only had a considerable protective effect against influenza A/H1N1 (47%; 95% CI 5–71%), but a similar effect was not observed against influenza A/H3N2 and B (6% in both cases) (Table 3).

IVE point estimates for current-season vaccination showed slight variability among seasons, ranging between 26 and 46% (Supplementary Table 5).

### Comparison of target population for influenza vaccination with and without COPD

A total of 6669 patients with major chronic conditions or aged ≥ 60 years were seen in primary care centres or hospitalized with ILI and tested for influenza during the study period. Among the controls, influenza vaccination coverage in the current season was higher in patients with COPD than in the rest the target population for influenza vaccination (68% vs. 59%, *p* < 0.001). The IVE in preventing influenza was evaluated according to the influenza vaccination status in 1761 (26%) COPD patients and 4908 (74%) patients without COPD. The effect of influenza vaccination in the current season did not differ significantly between COPD patients (aOR = 0.61; 95% CI 0.47–0.79) and target population without COPD (aOR = 0.60; 95% CI 0.51–0.69; *p*_comparison_ = 0.339), as well as the effect of influenza vaccination in prior seasons only (aOR = 0.75; 95% CI 0.53–1.08) and aOR = 0.64; 95% CI 0.52–0.79; *p*_comparison_ = 0.712), respectively. Results from sub-analyses in outpatients and inpatients were consistent (Table 4 and Supplementary Table 6).

**Table 4:** Effect of influenza vaccination status in the current and 5 prior seasons in preventing laboratory-confirmed influenza in the target population for influenza vaccination^a^ with and without chronic obstructive pulmonary disease.

	Cases/controls	Adjusted odds ratio (95% CI)^b^	*p* value
**All patients**			
Target population without COPD unvaccinated	776/866	1	
Target population without COPD vaccinated in prior seasons only	196/408	0.64 (0.52–0.79)	< 0.001
Target population without COPD vaccinated in the current season	833/1829	0.60 (0.51–0.69)	< 0.001
Patients with COPD unvaccinated	154/215	1	
Patients with COPD vaccinated in prior seasons only	79/172	0.75 (0.53–1.08)^c^	0.120
Patients with COPD vaccinated in the current season	309/832	0.61 (0.47–0.79)^c^	< 0.001
**Primary healthcare patients**			
Target population without COPD unvaccinated	348/167	1	
Target population without COPD vaccinated in prior seasons only	35/28	0.52 (0.30–0.91)	0.022
Target population without COPD vaccinated in the current season	169/161	0.49 (0.34–0.68)	< 0.001
Patients with COPD unvaccinated	54/30	1	
Patients with COPD vaccinated in prior seasons only	5/7	0.48 (0.13–1.75)^c^	0.264
Patients with COPD vaccinated in the current season	33/35	0.45 (0.22–0.92)^c^	0.029
**Hospital patients**			
Target population without COPD unvaccinated	428/699	1	
Target population without COPD vaccinated in prior seasons only	161/380	0.67 (0.53–0.84)	0.001
Target population without COPD vaccinated in the current season	664/1668	0.63 (0.53–0.74)	< 0.001
Patients with COPD unvaccinated	100/185	1	
Patients with COPD vaccinated in prior seasons only	74/165	0.78 (0.53–1.14)^c^	0.201
Patients with COPD vaccinated in the current season	276/797	0.63 (0.47–0.84) ^c^	0.002

*CI* confidence interval, *COPD* chronic obstructive pulmonary disease.

^a^Target population for influenza vaccination included people ≥ 60 years or with major chronic conditions.

^b^Odds ratio adjusted by age groups (25–64, 65–84 and ≥ 85 years), other major chronic conditions, healthcare setting (primary healthcare or hospital), and month-season of sample collection.

^c^Comparison of the adjusted odds ratios in COPD vs. non-COPD patients: all patients with current season vaccination, *p* = 0.339; all patients with vaccination in prior seasons only, *p* = 0.712; outpatients with current season vaccination, *p* = 0.468; outpatients with vaccination in prior seasons only, *p* = 0.745; inpatients with current season vaccination, *p* = 0.345; inpatients with vaccination in prior seasons only, *p* = 0.701.

### Impact of influenza vaccine coverage

**Figure 1:**
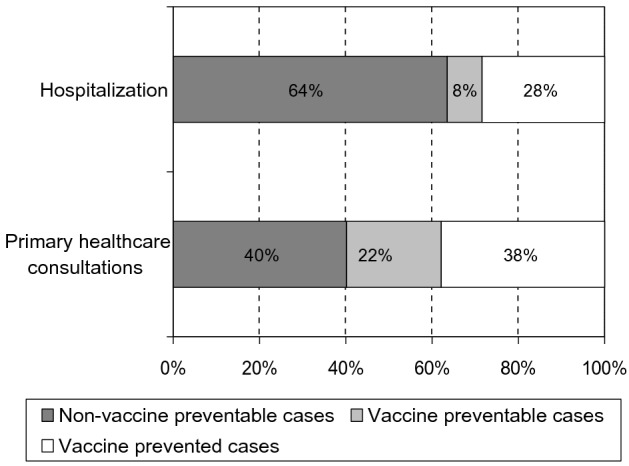
Impact of influenza vaccination in preventing primary healthcare consultations and hospitalizations for influenza among persons with chronic obstructive pulmonary disease.

The current-season influenza vaccination coverage in persons with COPD was 68% according to the control patients. Giving the IVE estimate and the vaccination status of cases, 38% of potential outpatient cases and 28% of potential hospitalizations with influenza in the study population have been prevented by influenza vaccination. We also estimated that other 35% of outpatient consultations and 11% of hospitalizations with confirmed influenza among patients with COPD (22% and 8% of the potential influenza cases, respectively) could be still prevented by extending the vaccine coverage (Fig. 1).

## Discussion

On average over five influenza seasons, influenza vaccination in the current season showed a moderate effect (40%) in preventing confirmed influenza in persons with COPD, and vaccination in prior seasons but not in the current one retained a possible low protective effect (24%). This residual protection appears to improve with vaccination in the current season. Both estimates seemed higher in preventing outpatient cases (60% and 58%, respectively) than hospitalized cases (37% and 19%, respectively), with no statistically significant differences between settings. Recent studies evaluated influenza vaccine effectiveness in preventing influenza hospitalizations in COPD patients, and reported similar results for current-season vaccination (38–48%)^14–16^. However, none of these studies had considered prior vaccination history or the effect in preventing primary healthcare consultations.

Our study suggest that the effect of current-season influenza vaccination was, on average of several seasons, moderate in preventing influenza A/H1N1 and B cases, and low for influenza A/H3N2, with a similar pattern that has been described in the general population^30^. Although with no statistically significant differences, the IVE estimates were slightly higher in individuals aged 25–64 years in comparison to older patients, which may be due to immunosenescence.

Influenza vaccination is recommended to all patients with high-risk conditions, including COPD. Thus, it is not ethically accepted to conduct placebo-controlled trials; and assessment of IVE should be based on observational studies^28^.

Since annual influenza vaccination is recommended to COPD patients, they may accumulate several doses of vaccines over the years. So far, no study had evaluated IVE considering the history of previous vaccinations in COPD patients; although vaccines received in prior seasons may preserve a significant protective effect in subsequent years^31–34^. We observed a considerable protective effect of the vaccines received in prior seasons in preventing influenza A/H1N1 cases (47%) among patients with COPD unvaccinated in the current season. However, this effect of vaccination history was low or null in most of the other analyses. Immunogenicity studies have shown that the level of antibodies peaks a few weeks after vaccination and then progressively declines, although it may persist for years^35^. On average, the annual drift of influenza A/H1N1 virus did not appear sufficient to evade the protective effect of the influenza vaccines from prior seasons^33^. However, the drift of influenza A/H3N2 and the alternate of lineages of influenza B may explain the lack of protection of prior season vaccination.

In our study, a similar IVE in preventing influenza was observed in target population for influenza vaccination with and without COPD. A recent study reported no differences in IVE in preventing hospitalizations among individuals with COPD and other comorbidities^14^. This suggests that patients with COPD have not worse response to influenza vaccination than patients with other comorbidities; thus, IVE estimates obtained for overall risk populations may be valid for COPD patients.

Among patients with COPD, co-diagnosis of asthma did not modify the IVE, as have been suggested in other study^14^.

The control group of the present study may be considered as a sample of the target population for influenza vaccination, and its analysis showed that the influenza vaccine coverage was higher among patients with COPD than among the rest. However, even in COPD patients in the present study, the vaccine coverage (68%) did not reach the goal of 75% proposed by the World Health Organization.

In the present study, around one in third potential influenza cases have been prevented by influenza vaccination, and some other cases and hospitalizations could still be prevented by extending the annual influenza vaccination to all patients with COPD. However, around half of outpatient consultations and hospitalizations for influenza among patients with COPD seems not preventable by using the influenza inactivated vaccination. High-dose influenza vaccines have demonstrated an appreciable higher IVE^36^; nevertheless, new more effective vaccines are necessary to achieve a decisive increase of the influenza vaccination impact.

This study has the following strengths. The test-negative design is the accepted reference design for IVE studies^28,30,37^. All cases were confirmed for influenza in the laboratory and controls were negative testers. Patients and doctors were blinded for the status of case or control, which improves comparability and reduces selection bias. Study individuals were recruited in the same region and influenza seasons, received the same vaccine brands, and were exposed to similar circulating viruses. The analyses over five influenza seasons increased the study size and achieved representation of different virus types and patient characteristics. The vaccination history was obtained from the regional vaccination registry^27^, and the study was limited to the population with stable residence in the region to avoid information biases. All analyses were adjusted for potential confounding variables^38^.

This study is also subject to limitations. COPD diagnosis was obtained from primary healthcare electronic records. Although some misclassification is possible, it would not likely have changed our results since IVE was similar in patients without COPD. Information on COPD severity was not available. This study was carried out in a single region where vaccination was recommended in people aged 60 years or over and in those with risk conditions, and only inactivated trivalent vaccine was used; therefore, care must be taken when generalizing our results to places with different indications for influenza vaccination, vaccination coverage, or where other types of vaccines are used. As the statistical power in some analyses was reduced, the results of a single season or in primary healthcare patients should be interpreted cautiously. The sample size was also limited to assess the incremental effect of the current season vaccination in persons with previous doses. The results of the pooled analyses of five influenza seasons should be considered as an average. Sex, pneumococcal vaccination, number of outpatient visits and hospitalizations in the preceding year have been described as potential confounding factors^15^, but their effect on the IVE estimates was ruled out in sensitivity analyses.

In summary, on average over five influenza seasons, the effect of current-season influenza vaccination was moderate to prevent confirmed influenza among patients with COPD, with no concluding difference in the effect between outpatient and inpatient cases. In people who were not vaccinated in the current season, influenza vaccines received in recent seasons may retain a residual protective effect in some situations. As influenza vaccination effect was similar in people with and without COPD, the estimates obtained for the target population may be valid for patients with COPD. An appreciable proportion of COPD exacerbations can still be prevented by increasing the influenza vaccine coverage. Our results reinforce the recommendations of annual vaccination for influenza in patients with COPD and highlight the urgent necessity of more effective vaccines.

## Supplementary Information

Supplementary Information.
