# Supplementary material for: Chronic obstructive pulmonary disease and influenza vaccination effect in preventing outpatient and inpatient influenza cases
Source: Sci Rep. 2022 Mar 22;12:4862. doi: 10.1038/s41598-022-08952-0 (PMC8940916; doi:10.1038/s41598-022-08952-0)
Supplement: Supplementary file 1 — Supplementary Information. [file 41598_2022_8952_MOESM1_ESM.docx]

**Supplementary Material**

**Supplementary Table 1.** Effect of influenza vaccination status in the current and 5 prior seasons in preventing laboratory-confirmed influenza among patients with chronic obstructive pulmonary disease

|  | **Cases / controls** | **Vaccination effect, % (95% CI)^a^** | **p-value** |
| --- | --- | --- | --- |
| ***Model 1*** |  |  |  |
| Never vaccinated | 154/215 | Reference |  |
| No current and 1 or 2 prior | 32/62 | 16 (–38 to 49) | 0.487 |
| No current and 3 to 5 prior | 47/110 | 28 (–11 to 54) | 0.132 |
| Vaccination in current season only | 12/36 | 51 (–1 to 76) | 0.053 |
| Current and 1 or 2 prior | 37/84 | 35 (–3 to 60)^b^ | 0.064 |
| Current and 3 to 5 prior | 260/712 | 40 (20 to 55)^b^ | 0.001 |
| ***Model 2*** |  |  |  |
| Never vaccinated | 154/215 | Reference |  |
| Vaccination in prior seasons only | 79/172 | 24 (–10 to 47) | 0.147 |
| Vaccination in current season only | 12/36 | 51 (–1 to 76) | 0.054 |
| Vaccination in current and prior seasons | 297/796 | 39 (19 to 54)^c^ | 0.001 |

CI: confidence interval.

^a^ Vaccination effect adjusted by age groups (25–64, 65–84 and ≥85 years), other major chronic conditions, healthcare setting (primary healthcare or hospital), and month-season of sample collection.

^b^ P=0.508 and p=0.566 for comparison with the category of vaccination in current season only with vaccination in current and 1 or 2 prior seasons and vaccination in current and 3 or 5 prior seasons, respectively.

^c^ P= 0.546 for comparison with the category of vaccination in current season only and vaccination in current and prior seasons.

**Supplementary Table 2.** Effect of influenza vaccination status in the current and 5 prior seasons in preventing laboratory-confirmed influenza among patients with chronic obstructive pulmonary disease by healthcare setting

|  | **Cases / controls** | **Vaccination effect, % (95% CI)^a^** | **p-value** |
| --- | --- | --- | --- |
| **Primary healthcare patients** |  |  |  |
| ***Model 1*** |  |  |  |
| Never vaccinated | 54/30 | Reference |  |
| No current and 1or 2 prior | 3/3 | 42 (–409 to 93) | 0.622 |
| No current and 3 to 5 prior | 2/4 | 69 (–125 to 96) | 0.249 |
| Vaccination in current season only | 2/3 | 65 (–183 to 96) | 0.325 |
| Current and 1 or 2 prior | 9/5 | 19 (–228 to 80)^b^ | 0.771 |
| Current and 3 to 5 prior | 22/27 | 72 (17 to 91)^b^ | 0.022 |
| ***Model 2*** |  |  |  |
| Never vaccinated | 54/30 | Reference |  |
| Vaccination in prior seasons only | 5/7 | 58 (–85 to 90) | 0.252 |
| Vaccination in current season only | 2/3 | 63 (–195 to 95) | 0.349 |
| Vaccination in current and prior seasons | 31/32 | 60 (–8 to 85)^c^ | 0.068 |
| **Hospital patients** |  |  |  |
| ***Model 1*** |  |  |  |
| Never vaccinated | 100/185 | Reference |  |
| No current and 1or 2 prior | 29/59 | 12 (–50 to 48) | 0.636 |
| No current and 3 to 5 prior | 45/106 | 23 (–21 to 51) | 0.255 |
| Vaccination in current season only | 10/33 | 49 (–10 to 77) | 0.085 |
| Current and 1 or 2 prior | 28/79 | 40 (–1 to 64)^d^ | 0.053 |
| Current and 3 to 5 prior | 238/685 | 36 (13 to 53)^d^ | 0.004 |
| ***Model 2*** |  |  |  |
| Never vaccinated | 100/185 | Reference |  |
| Vaccination in prior seasons only | 74/165 | 19 (–19 to 45) | 0.286 |
| Vaccination in current season only | 10/33 | 49 (–10 to 77) | 0.085 |
| Vaccination in current and prior seasons | 266/764 | 37 (14 to 53)^e^ | 0.003 |

Abbreviations: CI, confidence interval.

^a^ Vaccination effect adjusted by age groups (25–64, 65–84 and ≥85 years), other major chronic conditions, healthcare setting (primary healthcare or hospital), and month-season of sample collection.

^b^ P=0.463 and p=0.820 for comparison with the category of vaccination in current season only with vaccination in current and 1 or 2 prior seasons and vaccination in current and 3 or 5 prior seasons, respectively.

^c^ P=0.948 for comparison with the category of vaccination in current season only and vaccination in current and prior seasons.

^d^ P=0.700 and p=0.546 for comparison with the category of vaccination in current season only with vaccination in current and 1 or 2 prior seasons and vaccination in current and 3 or 5 prior seasons, respectively.

^e^ P=0.556 for comparison with the category of vaccination in current season only and vaccination in current and prior seasons.

**Supplementary Table 3.** Incremental effect of the current season vaccination in preventing laboratory-confirmed influenza among patients with chronic obstructive pulmonary disease who had been vaccinated in any prior season

|  | **Cases / controls** | **Vaccination effect, (95% CI) ^a^** | **p-value** |
| --- | --- | --- | --- |
| **All patients** |  |  |  |
| Vaccination in prior seasons only | 79/172 | Reference |  |
| Vaccination in current and prior seasons | 297/796 | 20 (–10 to 42) | 0.167 |
| **Primary healthcare patients** |  |  |  |
| Vaccination in prior seasons only | 5/7 | Reference |  |
| Vaccination in current and prior seasons | 31/32 | 5 (–359 to 80) | 0.947 |
| **Hospital patients** |  |  |  |
| Vaccination in prior seasons only | 74/165 | Reference |  |
| Vaccination in current and prior seasons | 266/764 | 22 (–9 to 43) | 0.144 |

CI: confidence interval.

^a^ Vaccination effect adjusted by age groups (25–64, 65–84 and ≥85 years), other major chronic conditions, healthcare setting (primary healthcare or hospital), and month-season of sample collection.

**Supplementary Table 4.** Sensitivity analyses of effect of influenza vaccination status in the current and 5 prior seasons in preventing laboratory-confirmed influenza among patients with chronic obstructive pulmonary disease

|  | **Cases / controls** | **Vaccination effect, (95% CI) ^a^** | **p-value** |
| --- | --- | --- | --- |
| **All patients** |  |  |  |
| Never vaccinated | 154/215 | Reference |  |
| Vaccination in prior seasons only | 79/172 | 15 (–25 to 43) | 0.404 |
| Current season vaccination | 309/832 | 34 (9 to 53) | 0.011 |
| **Primary healthcare patients** |  |  |  |
| Never vaccinated | 54/30 | Reference |  |
| Vaccination in prior seasons only | 5/7 | 33 (–218 to 86) | 0.615 |
| Current season vaccination | 33/35 | 42 (–98 to 83) | 0.386 |
| **Hospital patients** |  |  |  |
| Never vaccinated | 100/185 | Reference |  |
| Vaccination in prior seasons only | 74/165 | 10 (–36 to 41) | 0.611 |
| Current season vaccination | 276/797 | 31 (2 to 51) | 0.036 |

CI: confidence interval.

^a^ Vaccination effect adjusted by age groups (25–64, 65–84 and ≥85 years), other major chronic conditions, healthcare setting (primary healthcare or hospital), month-season of sample collection, sex, number of outpatient visits in the prior year, hospitalization within the prior 12 months, and pneumococcal vaccination.

**Supplementary Table 5.** Effect of influenza vaccination status in the current and 5 prior seasons in preventing laboratory-confirmed influenza among patients with chronic obstructive pulmonary disease by influenza season

|  | **Cases / controls** | **Vaccination effect, % (95% CI)^a^** | **p-value** |
| --- | --- | --- | --- |
| **2015-2016** |  |  |  |
| Never vaccinated | 30 /31 | Reference |  |
| Vaccination in prior seasons only | 13/19 | –20 (–216 to 55) | 0.716 |
| Current season vaccination | 37/100 | 45 (–12 to 73) | 0.099 |
| **2016-2017** |  |  |  |
| Never vaccinated | 24/33 | Reference |  |
| Vaccination in prior seasons only | 11/37 | 60 (–3 to 84) | 0.058 |
| Current season vaccination | 67/111 | 26 (–46 to 63) | 0.381 |
| **2017-2018** |  |  |  |
| Never vaccinated | 38/39 | Reference |  |
| Vaccination in prior seasons only | 29/39 | –7 (–117 to 48) | 0.858 |
| Current season vaccination | 94/164 | 41 (–4 to 66) | 0.070 |
| **2018-2019** |  |  |  |
| Never vaccinated | 38/55 | Reference |  |
| Vaccination in prior seasons only | 18/29 | –13 (–144 to 48) | 0.765 |
| Current season vaccination | 67/204 | 41 (–5 to 67) | 0.073 |
| **2019-2020** |  |  |  |
| Never vaccinated | 24/57 | Reference |  |
| Vaccination only in prior seasons | 8/48 | 57 (–11 to 83) | 0.082 |
| Current season vaccination | 44/253 | 46 (–4 to 72) | 0.063 |

CI: confidence interval.

^a^ Vaccination effect adjusted by age groups (25–64, 65–84 and ≥85 years), other major chronic conditions, healthcare setting (primary healthcare or hospital), and month of sample collection.

**Supplementary Table 6.** Adjusted odds ratio for laboratory-confirmed influenza in patients with chronic obstructive pulmonary disease (COPD) as compared to target population for influenza vaccination^a^ without COPD and similar influenza vaccination status in the current and 5 prior seasons

|  | **Cases / controls** | **Adjusted odds ratio (95% CI) ^b^** | **p-value** |
| --- | --- | --- | --- |
| **All patients** |  |  |  |
| Target population without COPD unvaccinated | 776/866 | 1 |  |
| Patients with COPD unvaccinated | 154/215 | 0.90 (0.71–1.16) | 0.416 |
| Target population without COPD vaccinated in prior seasons only | 196/408 | 1 |  |
| Patients with COPD vaccinated in prior seasons only | 79/172 | 1.06 (0.77–1.48) | 0.712 |
| Target population without COPD vaccinated in the current season | 833/1829 | 1 |  |
| Patients with COPD vaccinated in the current season | 309/832 | 0.92 (0.79–1.09) | 0.339 |
| **Primary healthcare patients** |  |  |  |
| Target population without COPD unvaccinated | 348/167 | 1 |  |
| Patients with COPD unvaccinated | 54/30 | 0.88 (0.52–1.49) | 0.646 |
| Target population without COPD vaccinated in prior seasons only | 35/28 | 1 |  |
| Patients with COPD vaccinated in prior seasons only | 5/7 | 0.80 (0.21–3.02) | 0.745 |
| Target population without COPD vaccinated in the current season | 169/161 | 1 |  |
| Patients with COPD vaccinated in the current season | 33/35 | 0.82 (0.47–1.41) | 0.468 |
| **Hospital patients** |  |  |  |
| Target population without COPD unvaccinated | 428/699 | 1 |  |
| Patients with COPD unvaccinated | 100/185 | 0.92 (0.69–1.22) | 0.569 |
| Target population without COPD vaccinated in prior seasons only | 161/380 | 1 |  |
| Patients with COPD vaccinated in prior seasons only | 74/165 | 1.07 (0.76–1.51) | 0.701 |
| Target population without COPD vaccinated in the current season | 664/1668 | 1 |  |
| Patients with COPD vaccinated in the current season | 276/797 | 0.92 (0.78–1.09) | 0.345 |

CI: confidence interval; COPD: chronic obstructive pulmonary disease.

^a^ Target population for influenza vaccination included people ≥60 years or with major chronic conditions.

^b^ Odds ratio adjusted by age groups (25–64, 65–84 and ≥85 years), other major chronic conditions, healthcare setting (primary healthcare or hospital), and month-season of sample collection.
